# The burden of Hepatitis B virus infection in Kenya: A systematic review and meta-analysis

**DOI:** 10.3389/fpubh.2023.986020

**Published:** 2023-01-26

**Authors:** Grace Naswa Makokha, Peiyi Zhang, C. Nelson Hayes, Elijah Songok, Kazuaki Chayama

**Affiliations:** ^1^Laboratory of Medical Innovation, Department of Collaborative Research, Graduate School of Biomedical and Health Sciences, Hiroshima University, Hiroshima, Japan; ^2^Department of Gastroenterology and Metabolism, Graduate School of Biomedical and Health Sciences, Hiroshima University, Hiroshima, Japan; ^3^Graduate School of Health Sciences, Kenya Medical Research Institute (KEMRI), Nairobi, Kenya

**Keywords:** Hepatitis B virus, Kenya, meta-analysis, review, prevalence

## Abstract

**Background:**

Chronic Hepatitis B virus (HBV) infection causes liver cirrhosis and cancer and is a major public health concern in Kenya. However, so far no systematic review and meta-analysis has been conducted to estimate the burden of disease in the country. A better understanding of HBV infection prevalence will help the government implement efficient strategies at eliminating the disease. This systematic review and meta-analysis was therefore conducted to summarize and update the available information on the burden of HBV in Kenya.

**Method:**

We systematically searched PubMed, Science Direct, Web of Science, Scopus, African Journals OnLine, and Google Scholar databases to retrieve primary studies conducted between January 1990 and June 2021 that assessed the prevalence of HBV infection in Kenya based on measurement of the Hepatitis B Surface Antigen (HBsAg). Meta-analysis was performed using the random effects model where HBsAg prevalence was estimated at a 95% confidence interval (CI) after simple pooling analysis. Potential sources of heterogeneity were also investigated.

**Results:**

Fifty studies were included in the meta-analysis with a sample size of 108448. The overall pooled prevalence estimate of HBV in Kenya was 7.8% (95% CI: 5.8–10.1). Subgroup analysis revealed the highest prevalence among patients presenting with jaundice at 41.7% (95% CI: 13.5–73.3) whereas blood donors had the lowest prevalence at 4.1% (95% CI: 2.4–6.3). Prevalence in Human Immunodeficiency Virus (HIV)-infected individuals was 8.2% (95% CI: 5.8–11.0). An estimate of the total variation between studies revealed substantial heterogeneity (I^2^ = 99%) which could be explained by the study type, the risk status of individuals, and the region of study.

**Conclusion:**

We present the first systematic review and meta-analysis of the prevalence of HBV in Kenya. Our results show that the burden of HBV in Kenya is still enormous. This calls for an urgent need to implement public health intervention measures and strategic policies that will bring the disease under control and lead to final elimination.

**Systematic review registration:**

https://www.crd.york.ac.uk/prospero/display_record.php?RecordID=264859, identifier: CRD42021264859.

## Introduction

Despite the existence of an effective vaccine, Hepatitis B virus (HBV) remains a major public health concern especially among developing countries (1). The World Health Organization (WHO) has emphasized the need for such countries to estimate their burden of viral infections and predict the global and regional economic impact thereof. Moreover, WHO launched a global program against hepatitis B and C infections whose aim by 2030 is to reduce the number of new cases of viral infections by 90%, reduce the number of hepatitis related deaths by 65, and treat 80% of viral hepatitis infections (2). However, it was emphasized that all countries commit to making sure all people have access to the tools needed to achieve this aim. The developing region of Africa comes second only after Asia in the burden of HBV chronic carrier rates with about 60 million infections (3). Countries in this region, including Kenya, have infection rates of >5% and account for 70% of all new HBV infections worldwide (4). Unfortunately, it has been difficult to determine the accurate burden of HBV in Africa due to under-reporting and poor record keeping. Besides, very few studies have been carried out and published to elucidate the occurrence of infections due to poor infrastructure and lack of funding. Nevertheless, the few available estimates show that there is a 60% lifetime risk of acquiring HBV infection in this regions (4).

HBV is a life-threatening infection that targets the liver and can result in acute and chronic disease. Whereas acute infection is self-limiting, chronic infection can cause liver disease and hepatocellular carcinoma (HCC) (5). According to the WHO, 10% of people infected with chronic HBV are diagnosed, out of whom only 22% receive treatment (6). This poses a huge challenge to developing countries such as those in Africa. The virus is estimated to account for 87 890 deaths annually in sub-Saharan Africa (3), but longitudinal studies on the incidence of cirrhosis in individuals in sub-Saharan Africa has proven difficult because liver biopsy is not a routine procedure, and non-invasive transient elastography is not readily accessible (7). However, numerous reports have shown the high incidence of HCC in sub-Saharan Africa, and 80% of cases are due to HBV infection (5, 8, 9). HCC is the second most common cause of death among cancer diseases globally and is not only a highly aggressive cancer, but the available treatment options are limited (10). Whereas HCC has many other risk factors, including hepatitis C virus (HCV) infection, aflatoxicosis, alcoholism, smoking, and hereditary conditions such as hemochromatosis, alpha-antitrypsin deficiency, tyrosinemia, anabolic steroids and estrogen levels (11–13), epidemiological and experimental studies demonstrate that chronic HBV infection is the major factor contributing to the development of primary liver cancer (13). For example, a high HCC incidence has been demonstrated in regions with a high seroprevalence for HBV infection (14). In addition, patients with HCC show 70–90% seroprevalence of HBV when compared to 10–20% HBV seroprevalence in the entire population in the same region (15). Additionally, a 10 to 100-fold risk of HCC has been observed in HBsAg carriers compared to non-carriers in different ethnic and social groups (5, 16).

Kenya, officially referred to as the Republic of Kenya, is a country in Eastern Africa. At 580, 367 square kilometers it is the world's 48th largest country by total area (17). The country is divided into eight provinces/regions, namely, Nairobi (the capital city), Central, Coast, Eastern, North Eastern, Nyanza, Rift Valley, and Western provinces (18). With a population of about 50 million people in the 2019 census, Kenya is the 29th most populous country and as at 2020, the third-largest economy in sub-Saharan Africa after Nigeria and South Africa, with a Human Development Index (HDI) of 0.555 (medium), ranked 145 out of 186 in the world (17). It is reported that 36.1% of Kenyans live on < US$1.90 per day (19). Despite major achievements in the health sector, Kenya still faces many challenges. For instance, the under-five mortality rate (U5M) remains high at 52 deaths per 1, 000 children as at 2014 (20) and only 43% of births were attended by a skilled health professional (21). The Hepatitis B vaccine was incorporated into the Kenya Expanded Program of Immunization (KEPI) in 2013 where it is given at 6, 10, and 14 weeks (22). The challenge is that the immunization programme only supports free vaccination for children up to 5 years of age but not people in other high-risk categories. A recent study involving several hospitals in Kenya demonstrated that chronic HBV infection was the leading cause of morbidity among patients with symptoms of liver disease seeking medical help (23). In terms of HBV genotypes, recent studies show genotype A as the predominant HBV genotype in Kenya, but genotype D and E have also been reported in the population, suggesting that HBV genetic diversity could be high in Kenya (24, 25).

Schweitzer et al. (26) published a systematic review and meta-analysis of the global prevalence of HBV with estimates by country where the pooled analysis estimate of the prevalence of HBV for countries in the African region stood at 8.8% (26). However, only eight studies were reported from Kenya, six of them published between 1957 and 1989, and two of them between 1990 and 2013. Moreover, further details on the source of the data presented were not provided, and the review gave an overall estimate of the prevalence, without emphasis on specific populations, especially at-risk groups to which interventions should be mostly directed. Therefore, the purpose of our review and meta-analysis is to provide an up-to-date and detailed summarization of the data on the prevalence of HBV in the general Kenyan population, as well as in specific populations such as blood donors, HIV patients, pregnant women, and healthcare workers, in particular. We strongly believe that such detailed data are necessary in order to spur decision-making toward interventions to curb the burden of HBV in Kenya.

## Methods

### Protocol and registration

The study was performed and reported according to the guidelines for Preferred Reporting Items for Systematic Reviews and Meta-Analyses (PRISMA) (27) (Supplementary File 1). The protocol used for the study was prospectively registered in the international database of prospectively registered systematic reviews with a health related outcome (PROSPERO) (28) under the registration number of CRD42021264859. As this is a systematic review and meta-analysis of published literature, ethical approval was not sought.

### Search strategy

We first searched previous systematic reviews and protocols related to the topic and setting using the PROSPERO database and the database of abstracts review of effects (DARE) to establish that no similar study has been previously registered or published. Peer reviewed articles published between January 1990 and June 2021 were comprehensively searched in PubMed, Science Direct, Web of Science, African Journals Online, Scopus and Google Scholar databases to identify all articles reporting the prevalence of HBV in Kenya. A manual search was also conducted for relevant articles as well as references therein. The keywords used were Hepatitis B, HBV, Hepatitis B surface antigen, HBsAg, prevalence, seroprevalence, Kenya, and Kenyan. The search terms above were used separately and in combination using Boolean operators “OR” and “AND.”

### Eligibility criteria

#### Inclusion criteria

Studies that reported the prevalence of HBV infection, were published in peer-reviewed journals in English language, were conducted in the country of Kenya, used the HBsAg test to diagnose HBV infection, and were published between January 1990 to 30 June 2021 were included in this review.

#### Exclusion criteria

Studies were excluded if they did not report the prevalence of HBV, if they were case reports, reviews, poster presentations, or editorials letters, or if they were published in non-English languages or were conducted outside the country of Kenya.

### Data collection and management

We used Mendel software to assemble and screen the articles, remove duplicates, and streamline the process of study selection. Two reviewers (GNM and ZP) independently screened the titles and abstracts followed by a full review of the text. Data from each study was extracted using the following variables: study characteristics (type of study, publication year, study location i.e., region/province, multi-region or national level), participant characteristics (age range, sex, year, and population group), prevalence of the HBV marker, type of laboratory test used, and number of participants the HBV marker prevalence was based on.

### Quality assessment

The Joanna Briggs Institute (JBI) critical appraisal checklist for studies reporting prevalence data was used to assess the overall methodological quality of included studies (29). The tool includes nine parameters with a response option of yes, no, unclear, and not applicable. The following parameters were evaluated: appropriateness of the sampling frame, proper sampling technique, adequacy of sample size, description of study subjects and setting, sufficient data analysis, validity of methods used for the identification of the condition, measurement for the condition in a standard and reliable manner, the appropriateness of statistical analysis, and adequacy of the response rate. A score of 1 was assigned for a “yes” response and 0 was assigned for “no” and ‘unclear' responses, where a total minimum score of 0 and a maximum score of 9 was assigned. The mean score was computed for each study. Two reviewers (GNM and ZP) independently conducted the quality assessment, and disagreements were resolved by consensus.

### Data synthesis and statistical analysis

The estimated prevalence of HBV was calculated as the number of HBsAg-positive subjects divided by the total number of subjects screened. The overall pooled and sub-group prevalence estimates were computed using a random effects model by the meta package of the statistical software R-version 4.0.2 (30). The test for heterogeneity was evaluated by the X^2^ test on Cochrane's Q statistic (ref), quantified by I^2^ value. The random-effects model was selected due to presence of significant heterogeneity (I^2^ > 50%). *P* < 0.05 was considered to indicate a statistically significant difference for all included studies. We assessed the sources of variation among studies with sub-group analysis using the following grouping variables: year of publication, study type, risk status, region, method of diagnosis, sample size and methodological quality. Sensitivity analysis was performed by removal of one study that had the largest sample size. Begg's funnel plot and Egger's weighted regression method were used to test for publication bias.

## Results

### Study selection

**Figure 1:**
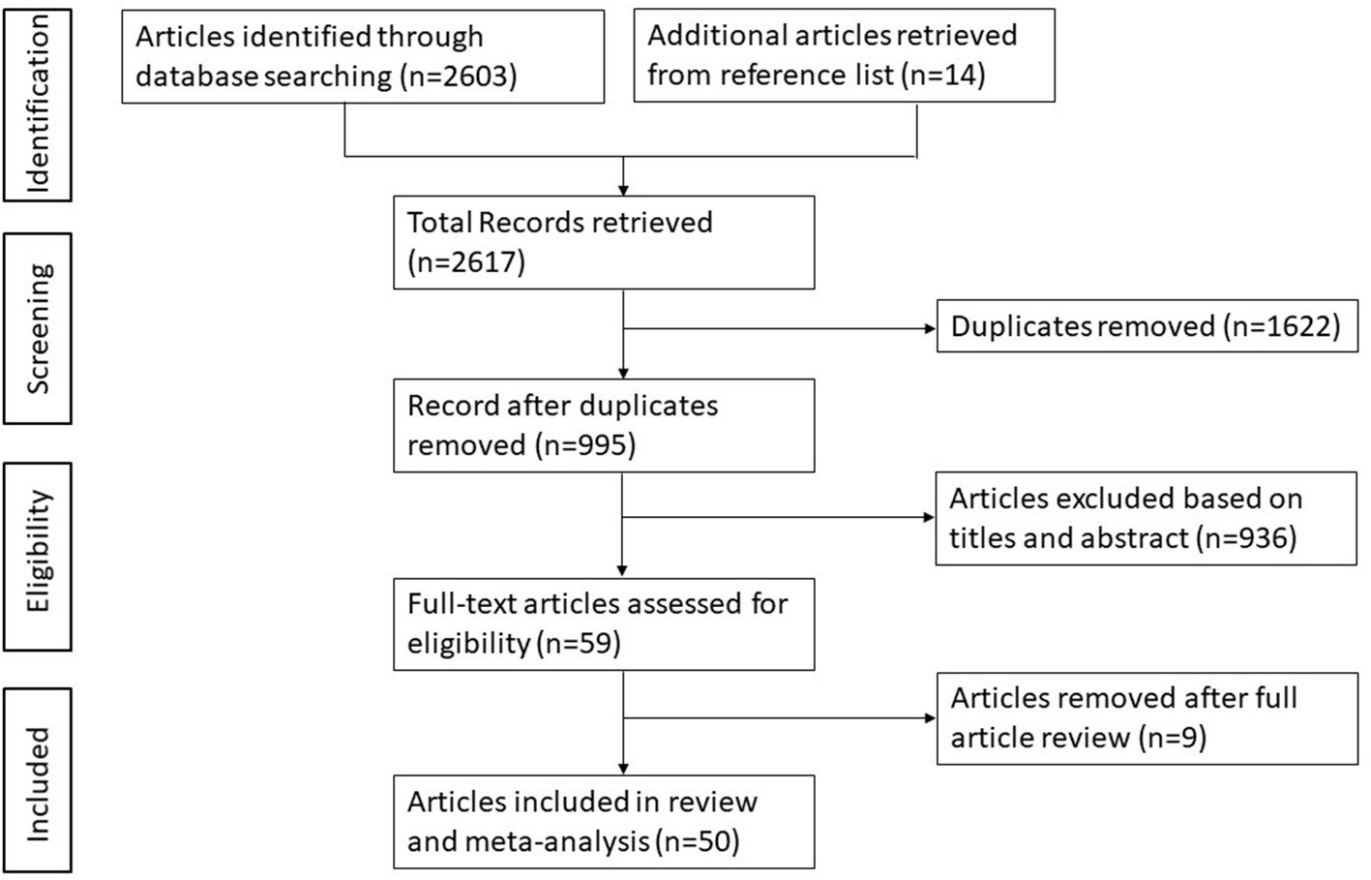
A flow diagram of the search results and study selection.

Studies were selected as shown in the PRISMA flow chart (Figure 1). Initially, a total 2,617 studies were identified through the six database searches and additional references. From these, 1,622 studies were removed due to duplication. The remaining 995 studies were screened based on title and abstracts, and 936 studies were excluded due to ineligibility. A further review of full articles was carried out on the remaining 59 studies, and 50 studies met the inclusion criteria to be used for the study.

### Characteristics of included studies

The characteristics of the included studies are summarized in Table 1. A total of 50 studies were enrolled in this systematic review and meta-analysis, with an overall sample size of 108,448. In terms of study design, most of the studies were cross-sectional (36, 72%) (22, 31–65), 6 each were prospective (23, 66–70) and retrospective (71–76), and one each for prospective cross-sectional (77) and retrospective cross-sectional (78). As for study populations, 19 studies involved HIV-infected individuals (31, 33, 40, 41, 43, 44, 47, 50, 52, 54, 55, 60, 64–70), 14 in other high risk groups [4 in liver disease patients (23, 35, 39, 77), 2 in drug users (46, 57), 2 in MSM (74, 76), 2 in healthcare/medical waste workers (53, 59), 1 study each for STI clinic attendees (51), kidney disease patients (34), and febrile patients (49), and 1 study in a combination of five high risk groups (22)]. As for the low-risk group, 10 studies were performed in blood donors (37, 48, 56, 58, 61, 62, 71, 73, 75, 78), 2 in pregnant women (36, 45), 2 in outpatients (32, 63), and 3 in a community setting (38, 42, 72). In terms of regional distribution, Nairobi had the highest number of studies at 16 (31, 33, 35, 37, 39, 40, 42, 45, 47, 51, 59, 61, 66, 67, 76, 77), followed by Rift Valley at 8 (22, 38, 44, 50, 52, 58, 63, 78), Coast at 7 (41, 43, 46, 49, 55, 68, 74), Nyanza at 6 (56, 62, 64, 70, 75, 77), Central 3 (32, 69, 73), and the Eastern region at 1 study (53). No study was reported from the North Eastern and Western regions and one study did not specify the location (71), whereas 8 studies were multi-regional or nationwide (23, 36, 48, 54, 57, 60, 65, 72). Gender-wise, 2 studies included men only (74, 76), 3 women only (36, 45, 68), and one children only (33). In terms of HBV diagnosis, majority of the studies (27, 54%) used ELISA as a method of detection (22, 23, 37, 40, 41, 44, 45, 48, 51–53, 55–60, 63, 66, 67, 69, 71, 73–76, 78), whereas reversed passive hemagglutination assay (RPHA) was used in 8 studies (31, 32, 34–36, 38, 39, 77), rapid diagnosis test (RDT) in 4 studies (46, 47, 54, 64), CMIA in 3 studies (33, 61, 68) and CLIA in 2 studies (50, 70). Only one study used polymerase chain reaction (PCR) as the method of detection (49). Most of the eligible studies were published in the year range of 2010–2019 (33, 66%), while the year ranges 2000–2009 and 2020–2021 had 7 studies each, and the 1990–1999 range had 3 studies (Supplementary Figure 2).

**Table 1:** Characteristic of studies included in the systematic review and meta-analysis of HBV in Kenya.

**Author**	**Publication Year**	**Study Design**	**Region**	**Population**	**Study Year**	**Age range/mean/ median**	**Sample size**	**HBsAg + (%)**	**Test Method**	**JBI Score**
Ogutu et al. (31)	1990	Cross-sectional	Nairobi	HIV-1+ Patients	NA	16–48	41	12.2	RPHA	6
Yamanaka et al. (32)	1991	Cross-sectional	Central	Outpatients	1986–1989	>0	8210	2.8	RPHA	8
Mwangi (71)	1999	Retrospective	N/A	Blood Donors	1995–1998	18–60	8762	4.3	ELISA	7
Chakraborty et al. (33)	2003	Cross-sectional	Nairobi	HIV-1+ Children	2000	1–18	54	4	CMIA	5
Otedo et al. (34)	2003	Cross-sectional	Nairobi	Kidney Patients	1998	44.3	100	8	RPHA	7
Atina et al. (35)	2004	Cross-sectional	Nairobi	Jaundice Patients	2000	0–67	84	13.1	RPHA	8
Otedo (77)	2004	Prospective cross-sectional	Nyanza	Jaundice Patients	2002–2003	7–76	519	64.4	RPHA	7
Okoth et al. (36)	2006	Cross-sectional	National	Pregnant Women	2001–2002	12–43	2241	9.3	RPHA	8
Harania et al. (66)	2008	Prospective	Nairobi	HIV-1+ Patients	NA	13–65	378	6.1	ELISA	7
Njeru et al. (37)	2009	Cross-sectional	Nairobi	Blood Donors	NA	16–54	400	2.3	ELISA	9
Kim et al. (67)	2011	Prospective	Nairobi	HIV-1+ Patients	2006–2008	36.5	389	6.9	ELISA	9
Mutuma et al. (38)	2011	Cross-sectional	Rift Valley	General Population	1994	>0	579	8.8	RPHA	8
Muchiri et al. (39)	2012	Cross-sectional	Nairobi	Acute hepatitis patients	2007–2008	16–83	100	3	RPHA	8
Day et al. (68)	2013	Prospective	Coast	HIV-1+ Women	2004–2010	33–41	159	7	CMIA	7
Irungu et al. (69)	2013	Prospective	Central	HIV-1+ Patients	2008–2010	>18	990	1.9	ELISA	9
Muriuki et al. (40)	2013	Cross-sectional	Nairobi	HIV-1+ Patients	2009	4–59	300	6	ELISA	8
Kerosi et al. (41)	2015	Cross-sectional	Coast	HIV-1+ IDUs	NA	33.2	91	14.3	ELISA	8
Kerubo et al. (42)	2015	Cross-sectional	Nairobi	General Population	2006–2007	15–54 (29.3)	1308	13.3	Mixed	8
Kibaya et al. (43)	2015	Cross-sectional	Coast	HIV-1+ IDUs	N/A	>18	72	12.5	Mixed	7
Wambani (44)	2015	Cross-sectional	Rift Valley	HIV-1+ Patients	2014	18–60 (43)	124	5.7	ELISA	8
Malungu Ngaira et al. (45)	2015	Cross-sectional	Nairobi	Pregnant Women	2014	16–49 (26.7)	287	3.8	ELISA	8
Kilongosi et al. (46)	2015	Cross-sectional	Coast	Drug Users	2010	18–55	558	4.5	Rapid Test	8
Ly et al. (72)	2016	Retrospective	National	General Population	2007	15–64	1091	2.1	Mixed	9
Mabeya et al. (47)	2016	Cross-sectional	Nairobi	HIV-1+ Patients	2015	33.4	400	5.5	Rapid Test	8
Maina et al. (50)	2016	Cross-sectional	Nairobi	HIV-1+ Patients	2015	>18	190	5.8	CLIA	8
Ngoi et al. (49)	2016	Cross-sectional	Coast	Febrile Patients	2014–2015	18–35	498	4	PCR	8
Nyairo et al. (48)	2016	Cross-sectional	National	Blood Donors	NA	NA	301	22.2	ELISA	6
Ochwoto et al. (23)	2016	Prospective	Multi-Region	Jaundice Patients	2012–2013	>15	332	50.6	ELISA	8
Greer et al. (70)	2016	Prospective	Nyanza	HIV-1 Patients	2007–2010	>18	105	11	CLIA	9
Wairimu et al. (73)	2016	Retrospective	Central	Blood Donors	2004	16–55	250	2.3	ELISA	8
Wahome et al. (74)	2016	Retrospective	Coast	MSM	2005–2014	18–49	369	6	ELISA	8
Gikunda and Karanja (51)	2017	Cross-sectional	Nairobi	STI clinic attendees	2014	32.8 (18–60)	200	9.5	ELISA	8
Okoth et al. (52)	2017	Cross-sectional	Rift Valley	HIV-1+ Patients	2009	39.3	247	9.7	ELISA	8
Wamamba et al. (75)	2017	Retrospective	Nyanza	Blood Donors	2015	30	2046	3.1	ELISA	8
Kisangau et al. (53)	2018	Cross-sectional	Eastern	Health Care Workers	2017	31 (19–67)	295	4.5	ELISA	9
Karoney et al. (78)	2018	Retrospective cross-sectional	Rift Valley	Blood Donors	2010–2012	16–60	68,404	1.1	ELISA	8
Too et al. (54)	2018	Cross-sectional	Multi-Region	HIV-1+ Patients	NA	>18	140	17.1	Rapid Test	6
Komu and Lwembe (55)	2018	Cross-sectional	Coast	HIV-1+ Patients	2015–2017	>18	446	4.9	ELISA	8
Onyango et al. (56)	2018	Cross-sectional	Nyanza	Blood Donors	2015–2016	16–65	1215	3.5	ELISA	9
Oyaro et al. (57)	2018	Cross-sectional	Multi-Region	Drug Users	2011–2012	>18	673	4.3	ELISA	8
Bartonjo et al. (58)	2019	Cross-sectional	Rift Valley	Blood Donors	2011–2012	16–65	594	5.6	ELISA	8
Kangethe (59)	2019	Cross-sectional	Nairobi	Medical Waste Handlers	NA	20–60 (41.5)	185	2.7	ELISA	8
Mwangi (60)	2019	Cross-sectional	National	HIV-1+ Patients	NA	>14	1829	29	ELISA	7
Aluora et al. (61)	2020	Cross-sectional	Nairobi	Blood Donors	NA	18–65	300	2.3	CMIA	8
Awili et al. (62)	2020	Cross-sectional	Nyanza	Blood Donors	2019	18–25	1000	3.4	Mixed	7
Jepkemei et al. (76)	2020	Retrospective	Nairobi	MSM	2009–2015	28	99	10.1	ELISA	7
Mercy Jelagat et al. (22)	2020	Cross-sectional	Rift Valley	Mixed High Risk Groups	2014–2016	35.2	860	10.7	ELISA	7
Koech et al. (63)	2020	Cross-sectional	Rift Valley	Outpatients	2015–2016	18–82 (35)	200	10	ELISA	6
Onyango et al. (64)	2021	Cross-sectional	Nyanza	HIV-1+ Patients	2018	3–76 (38.2)	225	6.2	Rapid Test	9
Salyani et al. (65)	2021	Cross-sectional	Multi-Region	HIV-1+ Patients	2019–2020	38.8	208	5.8	Mixed	7

### Methodological quality of included studies

The quality of the papers included in the study was assessed by two authors (GNM and ZP) using the Joanna Briggs Institute (JBI) Critical Appraisal Checklist for Studies Reporting Prevalence Data (Supplementary File 3). The studies scored a median of 8 (range 5–9) using JBI's nine items of risk bias. Five (10%) studies had a quality score of 6 or less, whereas the remaining 45 (90%) studies scored >6 (Table 1). All studies used valid diagnostic methods for HBV detection, and the prevalence was measured in a standardized way.

### Prevalence of HBV infection in Kenya

**Figure 2:**
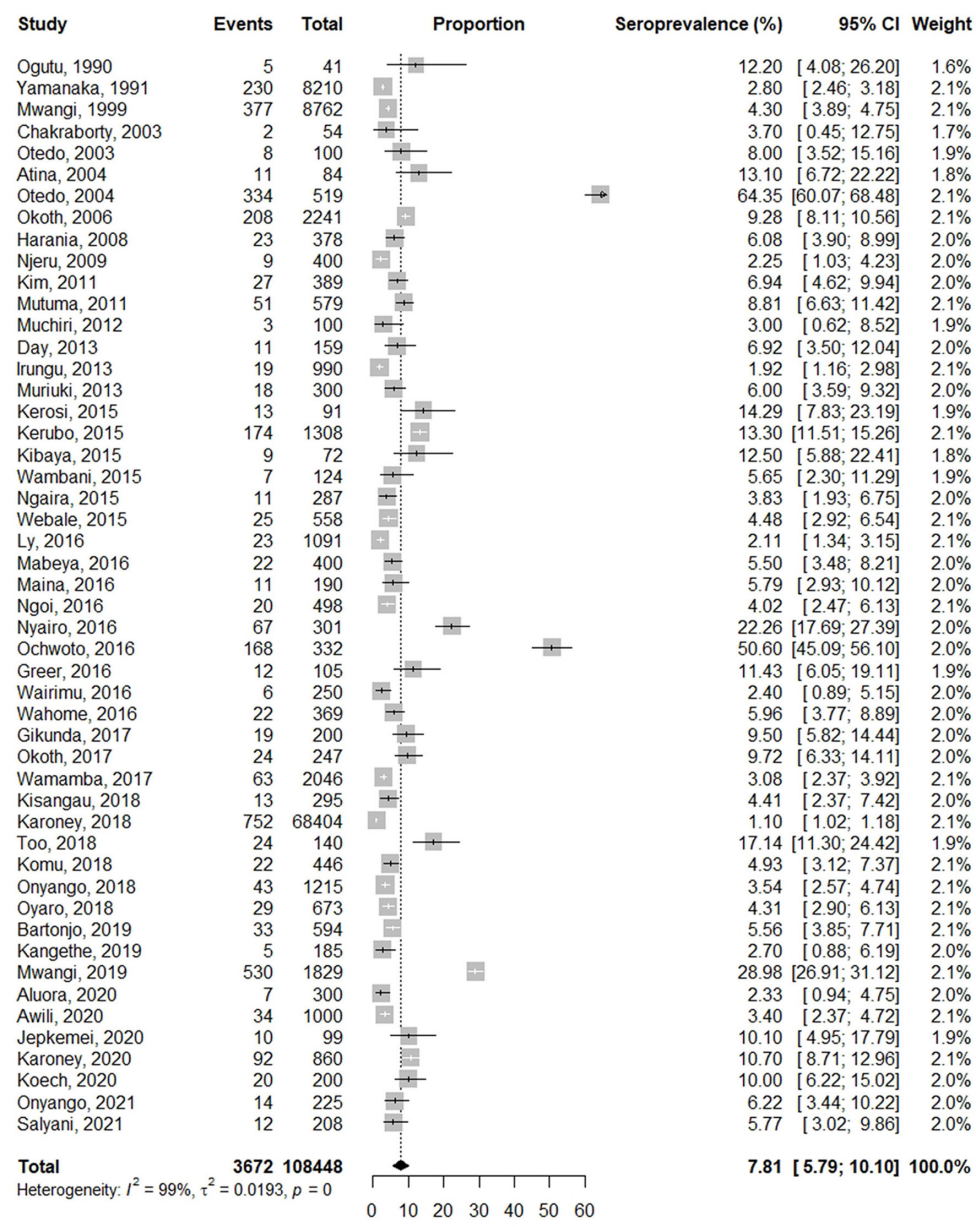
Analysis of the pooled prevalence estimate of HBV in Kenya.

The pooled prevalence of HBV infection was 7.8% (95% CI: 5.7–10.3), with a heterogeneity index of 99% (*P* < 0.0001) (Figure 2), which confirms a substantial heterogeneity among studies. The prevalence in the included studies ranged from 1.1% (blood donors) to 64.4% (jaundice patients). Out of the 50 studies, 19 studies (38%) reported a prevalence of equal to or more than 8%, categorized as high endemicity.

### Prevalence of HBV in blood donors, pregnant women, and jaundice patients

**Figure 3:**
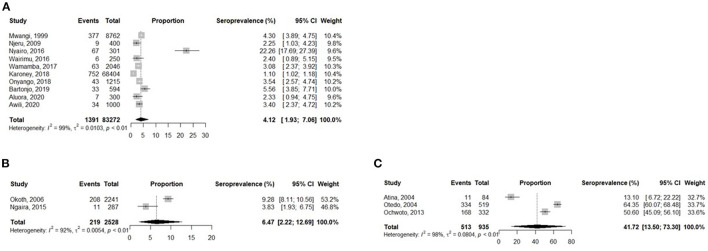
Analysis of the prevalence of HBV in Kenyan Blood Donors **(A)**, Pregnant women **(B)**, and Jaundice patients **(C)**.

Blood donors had a pooled prevalence of 4.1% (95% CI: 1.9–7.0), whereas jaundice patients reported a pooled prevalence of 41.7% (95% CI: 13.5–73.3) and pregnant women had a pooled prevalence of 6.5% (95% CI: 2.2–12.7) (Figure 3).

### Subgroup analysis of HBV prevalence

Table 2 presents the prevalence of HBV in various subgroups including assessment of heterogeneity and differences between subgroups. As expected, the prevalence of HBV was higher in high-risk populations at 9.5% (95% CI: 6.4–13.92) compared to their low risk counterparts at 5.1% (95% CI: 3.2–7.3). HIV patients had a pooled prevalence of 8.2% (95%CI: 5.8–11.0). Geographically, Nyanza province had the highest prevalence of 11.5% (95% CI: 1.1–30.3), whereas Central province had the lowest prevalence at 2.5% (95% CI: 1.9–3.1). In terms of sample size, studies with a sample size of < 1000 had a prevalence of 8.4% (95% CI: 5.9–11.4%), whereas those with sample sizes of >1000 had 5.8% (95% CI: 2.4–10.4). As per the method of diagnosis, RPHA accounted for the largest prevalence at 12.7% (95% CI: 3.6–26.0) while CMIA had the lowest at 3.9% (95% CI: 1.4–7.6). Considering the period of study, the year range of 2000–2009 had the highest prevalence at 12.4% (95% CI: 2.5–28.0), while the period of 1990–1999 reported the lowest prevalence at 4.5% (95% CI 1.4–5.8). Considering the methodological quality of studies, studies with a JBI score of < 6 had a pooled prevalence of 13.0% (95% CI: 7.3–20.0), whereas those with a score of ≥6 had a prevalence of 7.4% (95% CI: 5.2–10.0).

**Table 2:** Subgroup analysis of the studies reporting the prevalence of HBV in Kenya.

**Sub-group variables**	**Variable category**	**Included studies**	**Prevalence % (95% CI)**	**I^2^%**	***p*-heterogeneity**	***p-*difference**
HIV status	HIV	19	8.2 (5.8–11.0)	97.2	*p* < 0.01	0.7079
	Non-HIV	31	7.6 (4.6–11.3)	99.1	*P < 0.0001*	
Methodological quality	JBI ≤ 6	5	13.0 (7.3–20.0)	82.5	*p* < 0.001	0.0666
	JBI > 6	45	7.4 (5.2–10.0)	99.1	*p* < 0.001	
Region/Province	Nairobi	16	6.1 (4.4–8.0)	87.5	*p* < 0.01	< 0.0001
	Central	3	2.5 (1.9–3.1)	26.2	0.26	
	Unknown	1	4.3 (3.9–4.7)	NA	*NA*	
	Nyanza	6	11.5 (1.1–30.3)	99.5	*p* < 0.01	
	Multi-Region	8	15.0 (6.0–27.1)	99.2	*p* < 0.01	
	Rift Valley	8	6.6 (4.0–9.7)	98.3	*p* < 0.01	
	Coast	7	6.4 (4.2–8.8)	67.3	*p* < 0.01	
	Eastern	1	4.4 (2.3–7.1)	NA	NA	
Year of publication	1990-1999	3	4.5 (1.4–8.8)	94.0	*p* < 0.01	0.4978
	2000–2009	7	12.4 (2.5–28.0)	99.2	*p* < 0.01	
	2010–2019	33	7.5 (5.1–10.2)	99.0	< 0.0001	
	2020–2021	7	6.4 (4.0–9.4)	89.5	*p* < 0.01	
Method of diagnosis	RPHA	8	12.7 (3.6–26.0)	99.4	*p* < 0.01	0.1650
	ELISA	27	7.4 (4.8–10.6)	99.1	*p* < 0.001	
	CMIA	3	3.9 (1.4–7.6)	62.7	0.07	
	Mixed	5	6.4 (2.6–11.7)	97.3	*p* < 0.01	
	Rapid Test	4	7.5 (3.4–13.0)	85.8	*p* < 0.01	
	CLIA	2	8.1 (3.4–14.5)	65.0	0.18	
	PCR	1	4.0 (2.5–5.9)	NA	NA	
Risk Status	High	33	9.5 (6.4–13.2)	98.2	*p* < 0.001	0.0184
	Low	17	5.1 (3.2–7.3)	98.8	*p* < 0.001	
Sample size	≤ 1000	40	8.4 (5.9–11.4)	97.4	*p* < 0.001	0.2753
	> 1000	10	5.8 (2.4–10.4)	99.6	*p* < 0.001	
Study Type	Cross-sectional	36	7.2 (5.6–9.0)	97.3	*p* < 0.01	< 0.0001
	Retrospective	6	3.9 (2.4–5.7)	83.9	*p* < 0.01	
	Prospective cross-sectional	1	64.4 (60.2–68.4)	NA	NA	
	Prospective	6	11.3 (2.4–25.4)	98.8	*p* < 0.01	
	Retrospective cross-sectional	1	1.1 (1.0–1.2)	NA	NA	

### Publication bias

**Figure 4:**
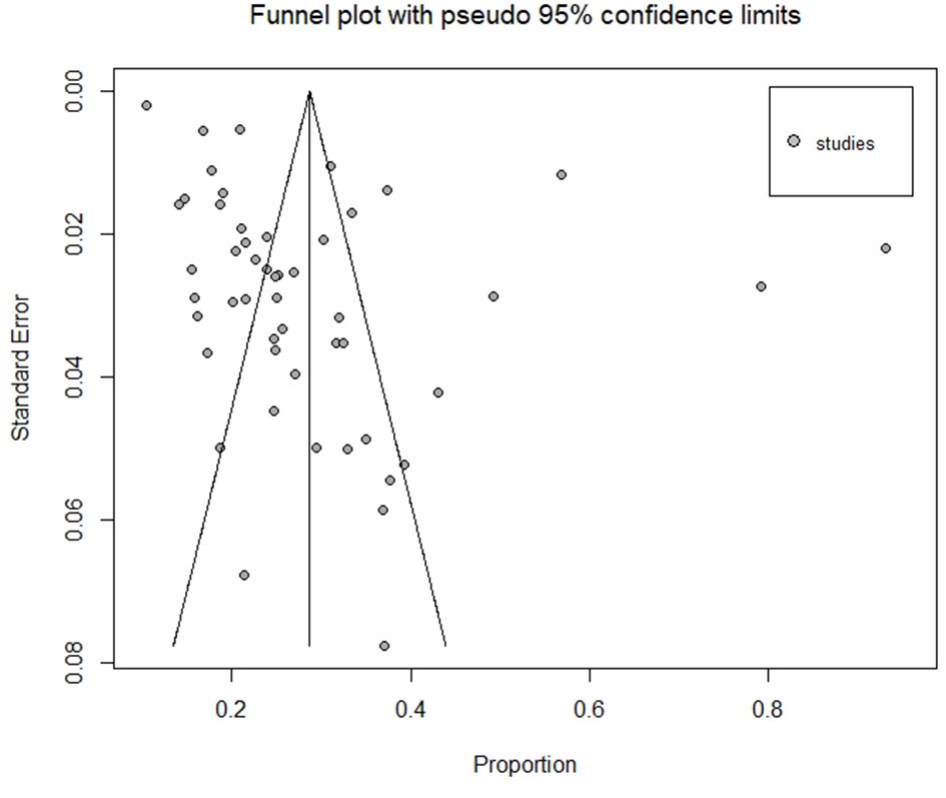
A bias assessment funnel plot of all included studies reporting HBV prevalence in Kenya.

The included studies were assessed for potential publication bias by Egger's test, which indicated publication bias (*p* < 0.0001). This was depicted graphically by a funnel plot comparing the effect size of each study (expressed as the proportion) on the x-axis vs. the standard error of proportion (on the y-axis) for the prevalence of HBV (Figure 4). The gray circles in the graph represent all studies included in the meta-analysis. The line in the center indicates the summary proportion and the other two lines represent the 95% confidence intervals. To facilitate the interpretation, the plot also includes the idealized funnel-shape that studies are expected to follow. Asymmetry about the pooled proportion line is consistent with the presence of publication bias.

### Sensitivity analysis

**Figure 5:**
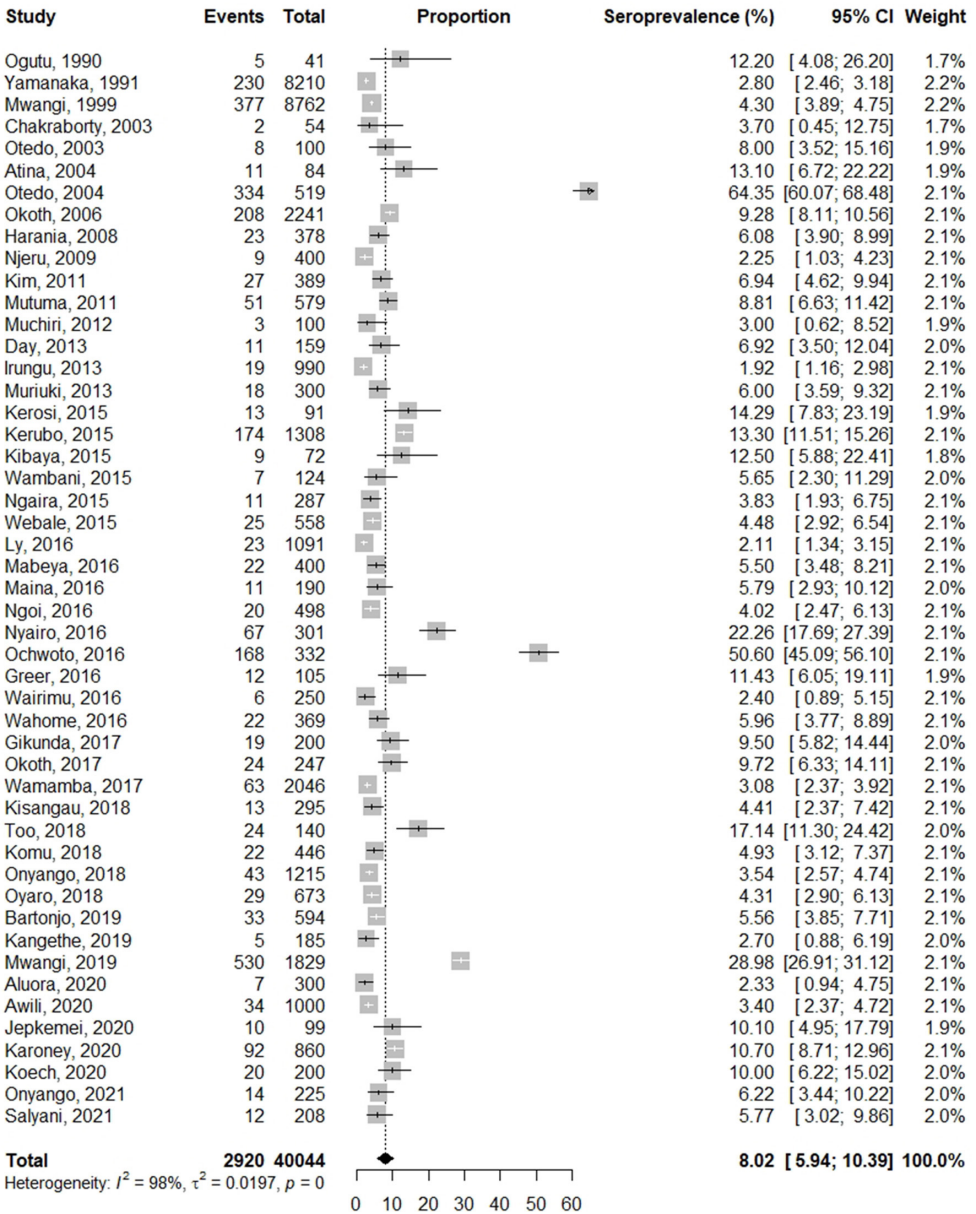
Sensitivity analysis in studies reporting prevalence of HBV in Kenya.

Due to the high heterogeneity of results, sensitivity analysis was done by omitting the study with the largest sample size (78) and assessing the effect based on the remaining studies. The overall pooled prevalence rate before omission was 7.8% (95% CI: 5.8–10.1) with a heterogeneity (I^2^) of 99%, *p* > 0.0001. After the omission, the pooled prevalence rate increased slightly to 8.0% (95%CI: 5.9–10.4) with a heterogeneity (I^2^) of 98%, *p* < 0.0001 (Figure 5). Thus, the calculated pooled prevalence was not affected by a single study, suggesting that the results are robust.

## Discussion

Kenya is one of the countries where no national review on the seroepidemiology of HBV has been published. We herein conducted a systematic review and meta-analysis covering the years 1990–2021 with the aim of assessing HBV prevalence in Kenya. We included 50 studies on HBV prevalence in different study populations of Kenya, with a total sample size of 108,448 and a pooled prevalence estimate of 7.8%. The study provides descriptive characteristics of HBV carriers in the general population, mainly blood donors, and in subgroups, such as pregnant women, drug users, people living with HIV, and healthcare workers. As a result of the expanded HBV vaccination program, the prevalence of HBV infection has decreased globally but remains highly endemic in some regions, including Africa (5). Going by the definition of HBV endemicity based on the HBsAg prevalence of low (< 2%), intermediate (2–7%), and high (>8%) (79), our results indicate high endemicity of HBV infection in Kenya. The information provided in this review and meta-analysis will contribute to improving knowledge of HBV infection epidemiology in Kenya and the larger Sub-Saharan Africa and promote effective policy-making.

The pooled prevalence of 7.8% in our study is considerably higher than the 2007 Kenya AIDS Indicator Survey that reported a prevalence of 2.1% among HIV-negative Kenyan adult and adolescent populations (72). The wide disparity could be explained by several factors. First and foremost is the small sample size of the study, as only 1,091 participants, among whom 27 tested positive for theHBsAg, were analyzed, relative to the current total population estimate of 50 million Kenyans (17). The researchers explained that a huge number of participants were excluded from the study due to insufficient amount of sera for testing. Secondly, despite the fact that Kenya has experienced one of the worst HIV epidemics (80), the study excluded HIV-positive individuals, even though they make up a large part of HBV infections due to the shared routes of transmission by the two viruses. Moreover, an estimate of 10% of HIV-infected persons are diagnosed with HBV worldwide (81). Therefore, inclusion of this population could have significantly altered the reported prevalence. The current prevalence is however closer to the one reported by a national serosurvey in our neighboring country of Uganda at 10.3%, which shares common cultural and traditional practices with Kenya, and involved a reasonable sample size (82), plus two other African countries have reported similar prevalences: Ghana at 8.4% (83), and Nigeria at 9.5% (84). These results imply that HBV continues to pose a significant burden in Kenya, as well as many other African countries, likely due to the weak healthcare systems on the continent. This calls for an urgent need to allocate enough funds to address the burden. On the other hand, Tanzania and Rwanda (85) have reported lower prevalence rates of 5.2 and 4.1% (85) respectively, likely due to the concerted efforts by healthcare workers and community members to improve HBV awareness in Tanzania, and the ongoing wide-scale vaccination campaign among people at high risk in Rwanda since 2015 (86, 87).

We found a high prevalence of HBV among HIV-infected individuals at 8.2%. A similar prevalence was reported in Ghana at 8.9% (83), and Nigeria at 9.9% (84), a rate higher than the WHO global estimate (6). This outcome suggests a high double disease burden for the affected individuals and increased risk for development of liver disease in the already immune-compromised patients. For instance, it has been reported that HIV infection may result in re-activation of “silent” chronic HBV and HIV-infected individuals have an increased likelihood of developing a chronic carrier state once infected with HBV (88). Moreover, a study in a Kenyan population showed that the level of non-responsiveness to the HBV vaccine was higher among HIV-1-infected participants compared with their HIV-1-uninfected counterparts at 35.8 vs. 14.3% (69). However, re-vaccination was effective in 86.3% of the HIV-1-infected initial non-responders, raising the overall response to 95% (69). It will therefore be necessary for the government to introduce routine hepatitis B testing and vaccination for HIV-1 infected patients, with increased focus on prevention and care for both viruses. In addition, enhanced determination of the immune response after vaccination in HIV-1–infected adults and timely re-vaccination of non-responders will increase the development of protective antibody titers in this high-risk population.

The studies in pregnant women showed a pooled prevalence of 6.5%, exactly the same as that reported by a recent meta-analysis in the West African country of Nigeria (89). This rate is, however, high compared to rates reported from the neighboring countries of Ethiopia at 4.8% (90), Rwanda at 3.7% (91), and Tanzania at 5.2% (92). Whereas the epidemiology of HBV in Kenya is not as well characterized, studies in the literature suggest that HBV infections occurring during early childhood *via* horizontal transmission and iatrogenic exposure account for the majority of HBV infections in Africa (93). Mother-to-child transmission (MTCT) is therefore presumed to be a minor mode of transmission in this region, unlike Asia (94). For instance in the Gambia, a west African country, only a small proportion (16.0%) of chronic carriers were infected *via* MTCT (95), and only 14.3% of HBV infected pregnant mothers were at risk for MTCT in an Ethiopian study (96). However, prevention of MTCT, which has been long neglected in the region is still important for many reasons. First, the annual number of infants perinatally infected with HBV is twice the number of incident pediatric HIV infections reported (*n* = 190 000) (94). Secondly, this mode of transmission increases the risk of chronicity following acute infection; 80–90%, 20-−0% and < 10% of individuals infected at birth, early childhood and adolescence/adulthood, respectively, develop chronic infection (94). Finally, MTCT may increase the risk of liver disease, including HCC, in those who become chronic carriers (94). We therefore recommend robust pre-conception screening and the implementation of “test and treat” interventions at low cost for infected mothers. Moreover, administration of Hepatitis B immune globulin to infants of infected mothers needs to be strengthened for the prevention of perinatal transmission of HBV infection.

Although we observed a lower prevalence of 4.1% among blood donors, this should raise an alarm concerning strict adherence to maintain a safe blood supply in the country, as 1 in 25 blood donors might be infected with HBV. The health systems should enhance control measures by including robust blood screening techniques on all blood donors and improve sensitization and awareness to the community at large regarding infection prevention measures for HBV and other transfusion-transmissible diseases such as HCV, HIV, and syphilis.

Despite advances in antiviral therapy, the primary prevention of HBV by vaccination is the gold measure of public health and the most cost-effective (97). Two studies evaluated factors affecting vaccine uptake in the Kenyan population, specifically the high-risk groups of health care workers and HIV-1 infected individuals (53, 98). In both studies, full vaccination rates remained low despite good knowledge of HBV infection and positive attitude toward vaccination. For instance, whereas vaccine uptake was reported at over 80% by both studies, only about 50 and 20% of vaccinated individuals, respectively were immune to the virus. It was concluded that the majority of individuals in both studies (> 80 %) only received one dose of the vaccine, hence, the effectiveness of vaccination was drastically reduced. On factors affecting vaccination status, both studies found that vaccine unavailability was the main hindrance followed by the unawareness on the safety and need for vaccination among the people. Lack of awareness was likely attributed to the fact that unlike HIV, HBV infection is not a program priority disease. The attention given to HBV and the level of funding is therefore low in Kenya as many other developing countries. There is therefore need to streamline vaccination programs especially for the high-risk groups. Moreover, the need to sensitize such populations on benefits of vaccines for the good of the communities within their reach should be enhanced. Key populations also play an important role in transmission dynamics of a number of infections and therefore constitute an essential partnership in prevention and control of HIV, HBV and HCV infections.

Generally, chronic HBV infection is ruled out in the absence of detectable HBsAg in the serum. However, the recent increased use of nucleic acid testing (NAT) to monitor HBV infection has led to the discovery of an HBsAg negative, HBV DNA positive phase of HBV known as occult HBV infection (OBI) (99). OBI has the following clinical implications: (i) HBV transmission by transfusion of blood products or by liver transplantation, (ii) HBV reactivation with consequent development of hepatitis B, as in cases undergoing potent immune-suppressive or chemotherapeutic therapies. Whereas, NAT for HBV has become a routine part of blood-donor infectious-disease screening in other parts of the world, developing countries such as Kenya still lag behind (100). In our review, only two out of the fifty studies examined HBV DNA by real-time PCR in the HBsAg negative population (65, 76). Both studies involved participants who were at high-risk for HBV and reported a prevalence of 19% of OBI among HBsAg negative individuals. This has serious implications in that out of every 10 people who test negative for HBsAg in the country, 2 have OBI and are at serious risk of transmission. Experts have recently recommended the use of highly sensitive nested PCR or real-time PCR assays that can detect fewer than 10 copies of HBV DNA for the screening and identification of OBI (101), especially in high-risk groups such as HIV, liver disease and hemodialysis patients. Such early detection and timely treatment reduces the risk of hospital cross-infection, and prevents health care infection and unnecessary medical disputes. Acknowledging the burden of OBI in Kenya will be necessary for the government to develop better national prevention and control measures.

Similar to the global trend of decreasing HBV prevalence (102), this study revealed a declining prevalence of HBV over time. Although the studies published between 1990 and 1999 had an average prevalence of 3.6%, those published thereafter from 2000 to 2009 rose sharply to 12.3%. However, in the subsequent years of 2010–2019 and 2020–2021, the prevalence dropped to 7.4% and further to 6.4%, respectively. This apparent drop in HBV prevalence over the recent years in Kenya can be partly explained by the rising effort of the ministry of health to improve, expand, and intensify immunization services in Kenya. Nevertheless, in order to further reduce the burden of HBV in Kenya, there is a need to increase the focus on vaccination of vulnerable groups, enhance prevention of mother to child transmission by identifying and offering treatment to HBV infected pregnant women, and implement full HBV vaccine coverage.

### Strengths

To the best of our knowledge, this is the first systematic review and meta-analysis that provides a pooled estimate of the prevalence of HBV in Kenya, both overall and among specific populations over the past three decades. Furthermore, the study employed a comprehensive search strategy across key data sources, and involved a large number of studies and study participants, covering most of the country's geopolitical regions. Finally, we were able to include many quality studies, as 90% of the included studies' methodologies were robust, hence they reflect the current situation in Kenya.

### Limitations

There are several limitations to this meta-analysis. First, the study includes only English language articles, which may complicate the interpretation of the results, as relevant studies in other languages may have been omitted. Secondly, only one study was performed in children (age range of 1–18 years and HIV-infected). This scarcity of data on the prevalence of HBV in infants meant that we could not provide an estimate prevalence in children under 5 years of age who are at high risk of MTCT. Thirdly, a major concern in meta-analysis studies is publication bias, which we detected in this study. Publication bias occurs when results of published studies are systematically different from results of unpublished studies (88). Many unpublished articles but with important findings may have been omitted which could seriously distort our reported estimate. Indeed, in our literature search, we came across many dissertations and theses with important information that never made it to publication in journals. This is common for research from many low and middle income countries, where registering every trial undertaken or publishing all studies is a challenge. Locating unpublished studies and unpublished outcomes of published studies for inclusion within a systematic review may provide a less biased estimate in future studies. Finally, we found a significant heterogeneity among the included studies, which might undermine confidence in the pooled estimate. Such high level heterogeneity calls for caution in interpretation of results not only in this paper but several other systematic reviews on HBV published from the larger Sub-Saharan Africa, with reports of I^2^ statistics of 94–99.9% (84, 89, 103, 104). However, we investigated the potential sources of heterogeneity, and the results showed that heterogeneity might have been due to expected differences in the study type, setting, and risk status.

## Conclusion

A very effective program for universal immunization was implemented in Kenya about three decades ago. However, based on our results, a large number of individuals who were born earlier remain infected since the burden of HBV infection in Kenya is high, with an uneven distribution among various sub-populations and geographical regions. Interventions targeting unvaccinated older individuals are expected to help reduce morbidity and mortality. Moreover, further studies are required to better understand the extent to which specific epidemiological factors might influence the regional distribution of HBV prevalence in Kenya. Finally, in order to achieve the WHO goal of HBV elimination in Kenya and the rest of the sub-Saharan Africa by 2030, there is urgent need for combined efforts in preventive and treatment strategies.

## Data availability statement

The original contributions presented in the study are included in the article/Supplementary material, further inquiries can be directed to the corresponding author.

## Author contributions

GM designed the study and wrote the paper. GM, ES, and PZ collected and analyzed the data. CH and GM revised the statistical analyses. CH and KC revised the paper. All authors approved the final version of the manuscript.

## Supplementary material

Supplementary File 1 The PRISMA checklist.

Supplementary Figure 2 The number of studies reporting prevalence in Kenyan per year range.

Supplementary File 3 The JBI's critical appraisal tools checklist.
