# Supplementary figures and images for: The burden of Hepatitis B virus infection in Kenya: A systematic review and meta-analysis
Source: Front Public Health. 2023 Jan 26;11:986020. doi: 10.3389/fpubh.2023.986020 (PMC9909240; doi:10.3389/fpubh.2023.986020)

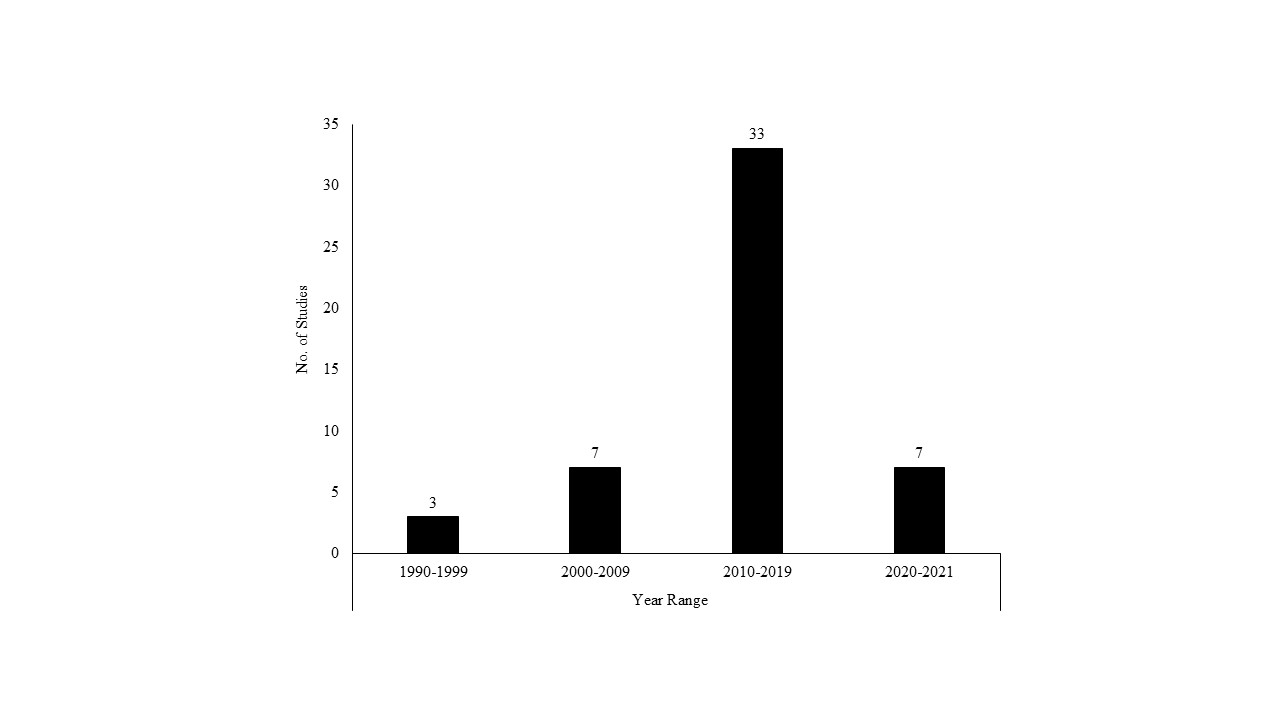

Supplement: Supplementary Figure 2 — The number of studies reporting prevalence in Kenyan per year range. [file Image_1.JPEG]
